# Effect of high intensity interval Nordic walking and strength training on selected biomarkers of metabolic syndrome in postmenopausal women with abdominal obesity: a quasi-experimental studies

**DOI:** 10.3389/fphys.2026.1841217

**Published:** 2026-06-19

**Authors:** Yangjun Liu, Hanxiao Xu, Wei Xie, Liying Liu, Monika Wiech, Zbigniew Ossowski

**Affiliations:** 1 School of Physical Education and Health, Chengdu University of Traditional Chinese Medicine, Chengdu, Sichuan, China; 2 Faculty of Physical Culture, Gdansk University of Physical Education and Sport, Gdańsk, Poland; 3 School of Acupuncture and Tuina, Chengdu University of Traditional Chinese Medicine, Chengdu, Sichuan, China

**Keywords:** abdominal obesity, high intensity interval Nordic walking, metabolic syndrome, postmenopausal women, strength training

## Abstract

**Objective:**

We aimed to evaluate the effects of a 12-week high intensity interval Nordic walking (HII-NW) and strength training (ST) intervention compared to standard lifestyle (CG) on selected obesity and metabolic syndrome (MetS) biomarkers in postmenopausal women with abdominal obesity.

**Methods:**

This study adopted a quasi-randomized controlled design, in which the experimental groups received HII-NW and ST interventions. The HII-NW intervention used a fast slow alternating walking pattern, maintaining the heart rate at 75%-85% of the maximum heart rate during fast walking and recovering to 60%-70% during slow walking, with each training session lasting 60 minutes and conducted 3 times a week. For ST, equipment such as dumbbells was used to perform resistance training on large muscle groups, with 3 sets of 8–12 repetitions for each exercise, 3 times a week, 45–60 minutes/session. The control group only received some daily life education training without any structured exercise intervention. The primary outcome measures included the changes in selected obesity and MetS biomarkers before and after the intervention in each group, as well as the differences in these measures between the intervention and control groups. Key metrics assessed were waist circumference (WC), blood pressure (BP), triglycerides (TG), high density lipoprotein cholesterol (HDL-C), and fasting blood glucose (FBG).

**Results:**

Compared to baseline, both the 12-week HII-NW and ST training significantly reduced WC, with the HII-NW group showing a reduction of 2.64 cm (p = 0.014) and the ST group showing a reduction of 2.83 cm (p = 0.011). Both training groups significantly increased HDL-C levels, with HII-NW showing an increase of 5.30 mg/dL (p = 0.007) and ST showing an increase of 3.34 mg/dL, (p = 0.016). HII-NW and ST training significantly lowered FBG levels, with reductions of 2.71 mg/dL (p = 0.019) and 2.22 mg/dL (p = 0.045), respectively. The HII-NW group also demonstrated a significant reduction in TG levels by 13.95 mg/dL (p = 0.027), along with notable improvements in abdominal obesity degree (AOD), percent body fat (PBF), body mass index (BMI), and heart rate HR. Compared to the control group, both HII-NW and ST significantly reduced FBG levels.

**Conclusion:**

This 12-week quasi-randomized controlled trial demonstrated that compared with baseline, both HII-NW and ST showed potential effects in improving obesity parameters (WC, PBF, BMI) and MetS markers (HDL-C, FBG) in postmenopausal women with abdominal obesity. Specifically, HII-NW significantly reduced TG levels and resting heart rate, while ST showed greater efficacy in reducing BMI. Compared with the control group, both interventions significantly decreased FBG levels. These findings highlight the potential of HII-NW and ST as targeted exercise strategies for metabolic health in this population, although long-term effects require further validation.

## Introduction

Postmenopausal women (PW), defined as females with permanent cessation of menstruation due to ovarian dysfunction (typically aged ≥45 years), are characterized by a precipitous decline in estrogen levels. As a key hormone regulating energy metabolism, estrogen deficiency drives the redistribution of adipose tissue from peripheral to abdominal regions, leading higher prevalence of abdominal obesity (waist circumference ≥80 cm) compared to premenopausal women (Kotronen et al., 2007; Lovejoy et al., 2008; Kelemen et al., 2010). Excessive abdominal fat accumulation directly exacerbates insulin resistance, increasing the risk of fasting glucose ≥6.1 mmol/L (Boldarine et al., 2020; Ko and Kim, 2020). Additionally, estrogen loss downregulates lipoprotein lipase activity, reducing triglyceride (TG) clearance, while inhibiting reverse cholesterol transport and elevating the incidence of high-density lipoprotein cholesterol (HDL-C) <1.3 mmol/L (Zhang et al., 2024). The weakened inhibitory effect of estrogen on the nuclear factor kappa-B (NF-κB) pathway further increases the risk of serum C-reactive protein ≥3 mg/L, exacerbating metabolic derangements (Moreau and Hildreth, 2014; Abd El-Kader and Al-Jiffri, 2019). Collectively, these mechanisms render PW significantly more susceptible to Metabolic Syndrome (MetS) than premenopausal populations (Marchi et al., 2017). This cohort not only faces heightened cardiovascular disease risk (El Khoudary et al., 2020) but also experiences progressive metabolic deterioration, while clinical interventions like hormone replacement therapy remain controversial due to adverse risks (Alexandersen et al., 2009), limiting their long-term use. Although exercise has been shown to improve insulin sensitivity (Remie et al., 2021), traditional exercise protocols lack stratification for PW’s hormonal decline, creating a critical evidence gap in precision exercise interventions. Exploring exercise modalities tailored to PW’s physiological characteristics is of urgent public health importance for MetS prevention and management.

Emerging evidence indicates that exercise interventions exhibit modality-specific effects on MetS, whereby traditional aerobic exercise enhances cardiorespiratory fitness but demonstrates limited targeting of abdominal obesity; conversely, isolated strength training (ST) increases muscle mass without rapidly optimizing glycolipid metabolic markers (Lesser et al., 2015; Nunes et al., 2016; Nunes et al., 2024). The high-intensity interval Nordic walking (HII-NW) examined in this study innovatively integrates the pole-supported walking pattern of traditional Nordic walking with the principles of interval training. Its biomechanical advantages include reduced lower-limb loading (Dziuba et al., 2015) and enhanced core muscle activation (Roy et al., 2020), which are beneficial for mitigating the muscle strength decline and balance impairment caused by estrogen loss (Cebula et al., 2020). Additionally, 30 minutes of high-intensity interval stimulation can significantly promote abdominal fat lipolysis (Maillard et al., 2018; Dupuit et al., 2020) and inhibit cortisol-induced metabolic dysregulation (Mohammadi et al., 2023). Compared with conventional modalities, HII-NW presents three key advantages: first, unlike traditional Nordic walking, it incorporates a high-intensity interval design to overcome the limitations of low intensity and insufficient metabolic stimulation; second, unlike standard HIIT, the pole-supported walking distributes lower-limb load and reduces knee joint stress, making it more suitable for PW; third, HII-NW achieves the combination of “low impact” and “high metabolic stimulation”, representing a novel exercise modality tailored specifically for abdominally obese PW. We hypothesized that HII-NW would be more effective in improving lipid profiles and cardiovascular-related outcomes. The ST protocol employs a progressive resistance regimen targeting major muscle groups to stimulate muscle fiber growth. This approach not only rapidly elevates energy expenditure (Nunes et al., 2016) and modulates neurometabolic pathways (Tedeschi, 2025), but also reduces metabolic risk and enhances insulin sensitivity (Nunes et al., 2024). For PW with abdominal obesity, resistance training-induced gains in muscle mass directly reverse metabolic impairments and interrupt the pathological cascade triggered by hormonal decline (Sá et al., 2023). We hypothesized that ST would exert more pronounced effects on improving BMI and body fat percentage.

Despite the widely recognized importance of exercise interventions, research specifically focused on PW remains insufficient. The theoretical rationale for comparing HII-NW and ST is threefold. First, regarding differences in metabolic pathways, HII-NW relies primarily on aerobic metabolism supplemented by anaerobic metabolism, and stimulates the AMPK/GLUT4 signaling pathway via high-intensity intervals to promote fat lipolysis and improve insulin sensitivity. In contrast, ST relies mainly on anaerobic metabolism and regulates glucolipid metabolism and reduces fat accumulation by increasing muscle mass. Their distinct metabolic mechanisms justify comparing their differential effects on MetS biomarkers. Second, in terms of physiological adaptation, HII-NW predominantly improves cardiopulmonary function, vascular endothelial function, and heart rate, thereby reducing cardiovascular disease risk. ST primarily increases muscle mass and strength to reverse menopausal muscle loss. These divergent adaptive targets enable them to improve different components of MetS. Third, in terms of training stimulus characteristics, HII-NW is a low-impact full-body exercise, while ST specifically enhances muscle mass. Both are safe and suitable for PW and may exert complementary effects. Comparing their benefits can support more precise clinical exercise prescription.

Accordingly, the present study addressed three key research questions:

1. What effects do 12 weeks of HII-NW and ST exert on obesity indices and MetS biomarkers in abdominally obese PW?
2. Are there any significant differences between these two exercise modalities?
3. What are the distinct target outcomes for each intervention?

We therefore proposed two hypotheses:

Hypothesis 1 (H1): Both HII-NW and ST will improve major obesity and MetS parameters in abdominally obese PW. Compared with ST, HII-NW will more effectively improve lipid metabolism (especially TG) and cardiovascular-related indicators (heart rate, HR) due to its combined advantages of low-impact Nordic walking and high metabolic stimulation from interval training.

Hypothesis 2 (H2): Compared with HII-NW, ST will more effectively reduce BMI and PBF in abdominally obese PW by promoting muscle mass gain through progressive resistance training and thereby enhancing basal metabolism.

Thus, this study utilizes a parallel controlled design with dual intervention groups to systematically evaluate the effects of HII-NW and ST on core MetS indicators WC, BMI and metabolic parameters, including BP, TG, HDL-C, Non-HDL, LDL-C, and FBG. We aim to address unexamined questions in current literature and provide valuable insights for novel exercise interventions targeting MetS in PW with abdominal obesity.

## Materials and methods

This study is reported following the CONSORT 2025 statement: updated guideline for reporting randomized trials.

### Study design and procedures

A pretest-posttest quasi-experimental design with repeated measures was employed to evaluate within-subject changes and between-group differences. The trial was conducted at the Gdansk University of Physical Education and Sport, Poland. Participants were recruited from the Third Age University (UTA) in Gdansk and Sopot, local clinics, and community noticeboards. From 193 eligible volunteers, 72 women aged 60–80 years were enrolled. All participants completed medical screening to confirm safe participation in physical activity. Cognitive function was assessed via face-to-face interviews including orientation, calculation, and memory tasks to ensure eligibility. Written informed consent was obtained from all participants in accordance with the Declaration of Helsinki (2018). The study protocol was approved by the Bioethics Committee of the Medical Chamber of Gdańsk (Decision No. KB-5/22). The 12-week intervention was conducted from March 20 to June 2022.

### Participants

This study enrolled PW with abdominal obesity and MetS risk factors. Postmenopausal status was defined as the absence of menstrual periods for at least 12 consecutive months. MetS was defined according to the International Diabetes Federation (IDF) criteria, which include five components: (1) waist circumference (WC) ≥ 80 cm; (2) BP ≥ 130/85 mmHg; (3) FBG ≥ 100 mg/dL; (4) TG ≥ 150 mg/dL; and (5) HDL-C < 50 mg/dL. Participants with two or more of these MetS components were included in the study.

Participants were excluded if they presented with uncontrolled hypertension, a history of oophorectomy, rheumatoid arthritis, pulmonary disease, type 2 diabetes requiring insulin therapy, or any contraindications to physical activity confirmed by comprehensive medical assessment. All participants reported medication changes via a standardized questionnaire to monitor and reduce confounding effects related to pharmacotherapy. Written informed consent was obtained from all participants prior to enrollment.

### Group allocation

**Figure 1:**
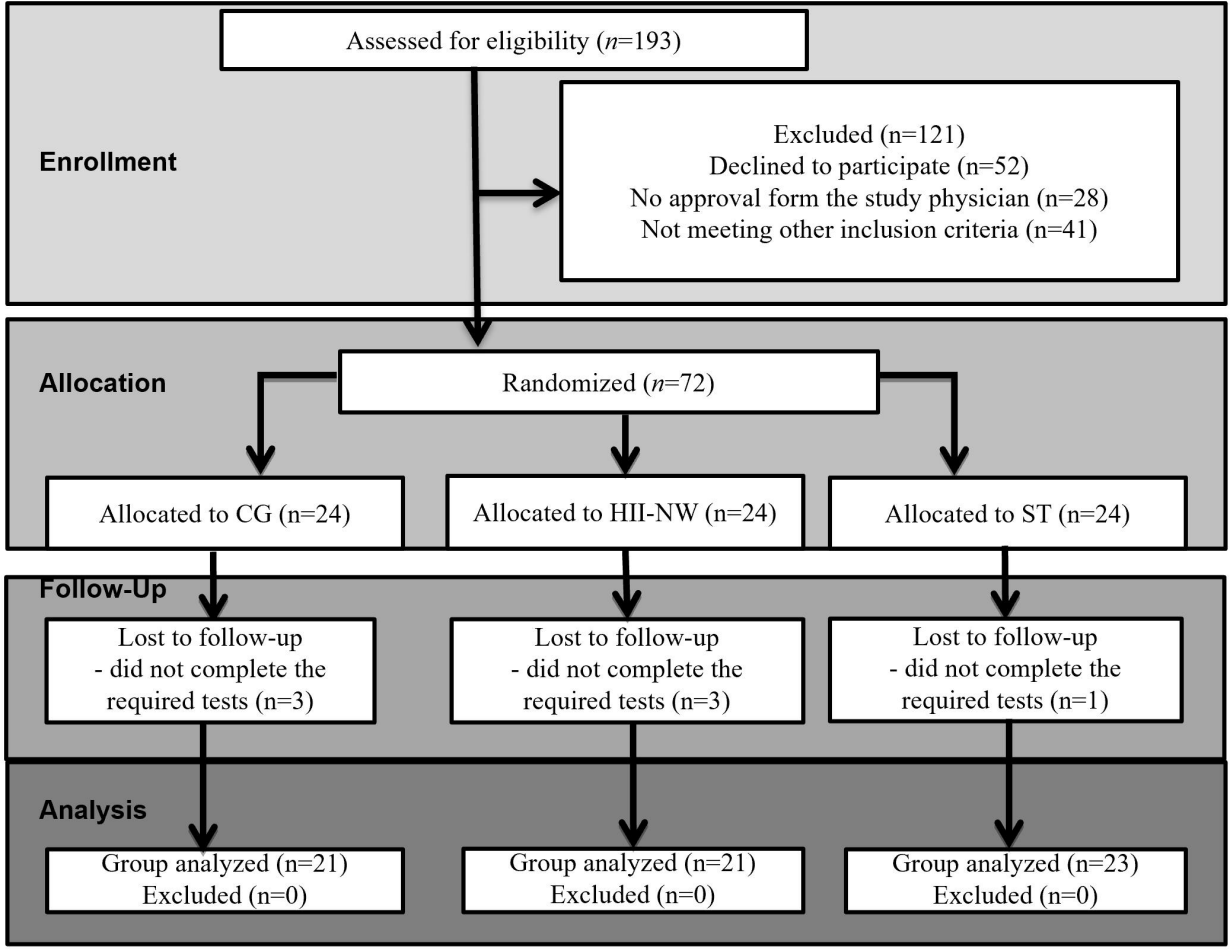
Recruitment process. HII-NW, High-Intensity Interval Nordic Walking; ST, Strength Training; CG, Control Group;.

A quasi-randomized design was used to assign participants to three groups in a 1:1:1 ratio. Grouping was performed by administrative residential districts to ensure baseline comparability of age, BMI, and living environment. If the number of participants in a single district was insufficient, recruitment was supplemented from adjacent areas with priority matching of baseline characteristics. The entire allocation process was conducted by independent statisticians with no involvement in intervention delivery to ensure transparency and objectivity. Participants were assigned to: (1) high-intensity interval Nordic walking (HII-NW) group; (2) strength training (ST) group; or (3) control group (CG). The detailed participant flow is presented in **Figure 1**.

### Compliance assessment

A dual verification system was used to evaluate intervention adherence, with compliance defined as completing ≥80% of scheduled training sessions. For the HII-NW group, compliance included meeting the session attendance rate and maintaining ≥85% of training duration within the target heart rate zone (75%–85% of age-predicted maximum heart rate; HRmax = 220 − age) (Gulati et al., 2010) monitored by Polar V-800 heart rate monitors. For the ST group, compliance required ≥80% attendance and proper exercise form verified by certified trainers. For the CG, compliance was defined as completing ≥80% of scheduled follow-up assessments.

Participant safety was monitored continuously by trained instructors before each session. Participants with three consecutive unexcused absences or overall compliance <80% were withdrawn from the study. Dropout reasons were documented, and all adverse events or health complaints were recorded and evaluated. Finally, 65 participants completed the study with an attrition rate of ≤12.5% and were included in the final statistical analysis.

### Intervention

This study compared HII-NW and ST based on their core differences in metabolic pathways, physiological adaptation, and training stimulus characteristics, aiming to clarify the differential effects of these two exercise modalities on MetS biomarkers in abdominally obese PW.

### HII-NW training protocol

**Figure 2:**
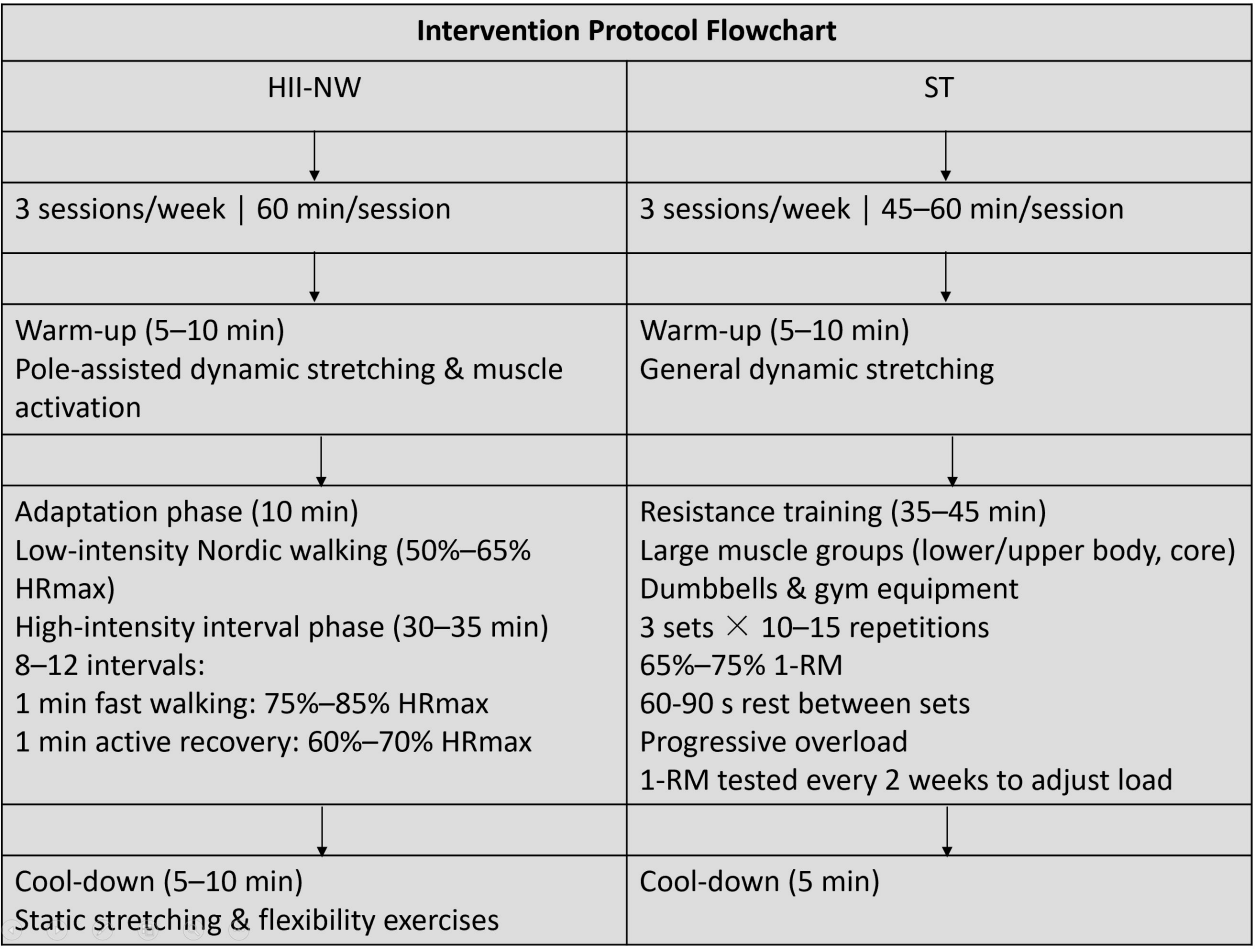
Intervention protocol flowchart for HII-NW and ST groups.

The HII-NW protocol (3 sessions/week, 60 min/session; **Figure 2**) was a novel, optimized modality tailored to the physiological characteristics of PW, distinct from traditional low-intensity continuous Nordic walking (NW) and high impact standard HIIT. All parameters (heart rate zones, interval duration) were validated in a preliminary pilot study to ensure safety and efficacy. The protocol consisted of four phases: Warm-up (5–10 min), Adaptation phase (10 min), High-intensity interval phase (30–35 min), Cool-down (5–10 min). Training intensity was continuously monitored using Polar V-800 heart rate monitors (Polar Electro Oy, Finland), with real-time HR data recorded to ensure adherence to target intensity zones (60%–85% HRmax) throughout the 12-week intervention.

### ST protocol

The ST program (3 sessions/week, 45–60 min/session; **Figure 2**) was delivered over 12 weeks in gyms and sports halls, focusing on progressive overload of major muscle groups (lower/upper body, core): Warm-up (5–10 min), general dynamic stretching to prepare for resistance training; Resistance training (35–45 min), Gym-based training (2 sessions/week), Integrated muscle group exercises using HES multi-station gym equipment (A-25-Sk, Wrocław); Sports hall training (1 session/week): Free weight exercises (dumbbell squats, presses, curls, deadlifts); Cool-down (5 min). The program aimed to comprehensively strengthen arm, leg, core, abdominal, and chest muscles.

### Control group

Participants in the CG maintained their habitual daily activities without engaging in any structured physical training throughout the study period.

### Medication

Participants were also required to disclose information regarding any prescription medications; those on medication were under the continuous supervision of medical experts to monitor their dosages. To minimize potential confounding variables, the types and doses of medications were kept constant throughout the study.

### Outcome measurement

Data regarding participants’ demographics and health information, including age, ethnicity, educational attainment, smoking history, as well as medication use for diabetes, hypertension, and dyslipidemia, were collected through surveys. All outcomes were measured both before and after the intervention. The primary and secondary outcomes are outlined below.

### Primary outcomes

#### Waist circumference

WC was assessed using a plastic measuring tape (HOLTAINR, Crosswell, UK), measured at the midpoint between the highest point of the iliac crest and the lower margin of the 12th rib. Care was taken to ensure that the measuring tape remained horizontal and laid flat against the skin without compressing it (Panoulas et al., 2008).

#### Activity time per week and average daily steps

Continuous monitoring was performed using the Polar V-800 heart rate monitor (Polar Electro Oy, Finland), which automatically recorded daily physical activity duration and step counts. Researchers aggregated weekly data through the device’s proprietary software to calculate average daily steps and total weekly activity time.

#### Hemodynamics

BP measurements were taken at specific time points during the study. Resting systolic blood pressure (SBP) and diastolic blood pressure (DBP) were recorded using an Omron HEM-907XL device (Omron Healthcare, Illinois, USA). The results were expressed in millimeters of mercury (mmHg). Participants rested in a seated position for 5 minutes prior to taking three measurements, with a 1-minute interval between assessments. The average of three measurements was utilized to calculate both SBP and DBP.

#### Blood sample collection and analysis

To eliminate the effects of acute physical exercise on lipid and glucose levels, blood samples were collected in the morning under fasting conditions for laboratory analysis, specifically two days before the start of the training program and two days after its completion. Biochemical blood analyses were performed in an accredited diagnostic laboratory, with all participants undergoing 12-hour fasting blood collection. FBG, TG, TC, and concentrations of HDL-C and LDL-C were measured using a Cobas 6000 c501 automated biochemistry analyzer (Roche Diagnostics, Germany). All analyses were conducted according to the manufacturer’s protocols using enzymatic colorimetric (spectrophotometric) methods and dedicated diagnostic kits. Each measurement was performed in duplicate and analyzed by qualified laboratory personnel to ensure the highest quality and reliability of results.

### Secondary outcomes

#### Height

Measurements of height was recorded with an accuracy of 0.1 centimeters. Height was assessed using a portable Harpenden stadiometer (model 98.603, Crosswell, UK), with participants required to stand barefoot in an upright position during measurement.

#### Weight and obesity indices

Weight (BM), visceral fat area (VFA), and body fat percentage (PBF) were evaluated using the Octapole Bioimpedance InBody 720 analyzer (Biospace, Seoul, South Korea) according to standard procedures. While DEXA is considered a gold standard, the InBody 720 shows a high correlation (r > 0.9) with DEXA measurements in populations with similar anthropometric characteristics, and has the advantages of high reproducibility and low measurement error (standard error approximately 2-3%). It has been validated in previous studies (Ogawa et al., 2011) for its reliability in estimating body composition parameters, including PBF and VFA, in postmenopausal populations. During the assessment, participants were required to be barefoot and dressed in light clothing. The analyzer employs eight contact electrodes placed on specific body parts at varying frequencies (1, 5, 50, 250, 500, and 1,000 kHz) to evaluate body composition. This device allows for independent analysis of five basic body segments. BMI was calculated as BM/height² (kg/m²).

#### Sample size calculation

Sample size calculation was performed using G*Power 3.1 software based on the primary outcome measure (WC). A one-way ANOVA (F-test, fixed effects) was applied for three independent groups (HII-NW, ST, and CG). A moderate effect size (f = 0.25), a significance level (α = 0.05), and a statistical power (1−β = 0.80) were set according to previous relevant studies (Lera Orsatti et al., 2014). The key parameters for the calculation are summarized in **Table 1**.

**Table 1:** Key parameters for sample size calculation (G*Power 3.1).

Parameter	Value
Statistical test	F-test, one-way ANOVA (fixed effects, omnibus)
Primary outcome	WC
Number of groups	3 (HII-NW, ST, CG)
Effect size (f)	0.25 (moderate)
α level (Type I error)	0.05
Power (1−β)	0.80
Required sample size per group	22
Dropout rate	10%–15%
Final recruited per group	24

The calculation indicated that 22 participants per group were required. To account for an expected 10–15% attrition rate during the 12-week intervention, the initial sample size was increased to 24 participants per group to ensure adequate statistical power for the final analysis.

### Statistical analysis

All data were presented as mean ± standard deviation (SD) to describe the variability within the sample, as it directly reflects the dispersion of individual data points around the mean. Data were analyzed using the Windows version of IBM SPSS Statistics, version 26.0 (Chicago, Illinois, USA). The normality of the data was evaluated using the Shapiro-Wilk test. For comparisons among the three groups, a one - way analysis of variance (One - way ANOVA) was conducted as the F - test when the data were normally distributed. Following a significant F - test result, Tukey’s Honest Significant Difference (HSD) test was applied for pairwise comparisons. In cases where the data did not meet the normality assumption, the Kruskal-Wallis rank sum test was used for overall group comparison, and the Mann-Whitney U test was employed for pairwise comparisons with Bonferroni correction (α = 0.05/3). For between group comparisons of two groups, independent t - tests were used when data were normally distributed; otherwise, the Mann-Whitney U test was applied. For within group analyses, paired sample t - tests were conducted for normally distributed data, while the Wilcoxon signed - rank test was utilized for non - normal data. Before analyzing the intervention effects, baseline characteristics of the three study groups were tested for balance. One-way analysis of variance (ANOVA) was used to compare differences in baseline variables, including age, obesity indices, and MetS related indicators, among the HII-NW group, ST group, and control group. A p - value of <0.05 (two - tailed) was considered statistically significant.

## Results

### Sample characteristics

Seventy-two participants were quasi-randomly assigned to three groups, with 65 completing the study. Baseline characteristics of the completers (HII-NW: 21 participants, ST: 23 participants, CG: 21 participants) were as follows:(1) Age: HII-NW (70.10 ± 4.23 years), ST (67.65 ± 4.23 years), CG (70.00 ± 3.02 years; p = 0.99). (2) Obesity indices: 70.8% overweight (BMI ≥25 kg/m²), 24.6% obese (BMI ≥30 kg/m²). All had WC >80 cm (95.07 ± 9.29 cm in HII-NW, 94.89 ± 11.07 cm in ST, 96.38 ± 11.93 cm in CG; p = 0.93). (3) MetS: 72.3% had elevated BP, 53.9% elevated FBG, 32.3% elevated TG, and 38.5% low HDL-C. 63.1% met criteria for MetS (≥3 risk factors). The compliance rates were 85% in the HII-NW group, 82% in the ST group, and 90% in the CG. There was no significant difference in compliance rates among the groups (p = 0.68). Baseline demographic, clinical parameters, and compliance rates did not differ significantly between groups (**Table 2**), and no adverse events were reported.

**Table 2:** The baseline demographic and clinical characteristics of participants.

Characteristics	HII-NW Group (n=21)	ST Group (n=23)	CG Group (n=21)	*P*
Age, years	70.10±4.23	67. 65±4.23	70.00 ±3.02	*0.99*
Height, cm	159.71±6.15	159.78±6.14	159.90±5.61	*0.07*
BMR, kcal/d	1301.45±103.80	1298.36±97.57	1307.57±92.99	*0.69*
PBF, %	37.39±5.71	36.39±6.76	39.96±7.69	*0.21*
AOD	0.93±0.05	0.94±0.05	0.94±0.06	*0.86*
BMI, kg/m²	27.16±3.24	26.77±3.88	29.00±4.45	*0.14*
ACT, min / w	318.70±107.65	341.56±90.11	344.73±86.52	*0.36*
ANS, steps/day	10274.76±3755.74	11201.89±2943.00	11538.47±4476.73	*0.41*
WC, cm	95.07±9.29	94.89±11.07	96.38±11.93	*0.93*
SBP, mmHg	131.76±18.02	139.94±16.24	134.62±19.18	*0.35*
DBP, mmHg	81.54±8.54	85.74±8.07	82.19±12.14	*0.31*
HR, bpm	79.24±13.19	79.99±11.44	80.29±10.28	*0.96*
TG, mg/dl	129.86±42.71	122.91±39.60	141.24±64.83	*0.88*
HDL-C, mg/dl	55.79±11.71	56.36±11.61	53.17±12.08	*0.72*
NO-HDL-C, mg/dl	164.40±44.53	161.97±40.16	166.54±37.22	*0.93*
LDL-C, mg/dl	138.43±39.36	133.00±39.76	138.33±36.52	*0.87*
FBG, mg/dl	101.19±11.08	102.09±9.57	115.33±42.49	*0.71*

Data are expressed as mean (± SD) or percentage as appropriate (%).

HII-NW, High-Intensity Interval Nordic Walking; ST, Strength Training; CG, Control Group; BMR, Basal Metabolic Rate; PBF, Percent Body Fat; AOD, Abdominal Obesity Degree; ACT, activity time per week [min]; ANS, Average number of steps per day; BMI, Body mass index; WC, Waist circumference; SBP, Systolic blood pressure; DBP, Diastolic blood pressure; HR, Heart rate; TG, Triglycerides; HDL-C, High Density Lipoprotein Cholesterol; NO-HDL-C, non-High Density Lipoprotein Cholesterol; LDL-C, Low Density Lipoprotein Cholesterol; FBG, Fasting blood glucose.

### Primary outcome

**Table 3** presents the variations in selected primary outcome indicators among the three groups both pre- and post- training, as well as reflecting the inter group comparison results following the testing. Comparisons between the HII-NW and ST groups before and after training revealed significant reductions in WC, with the HII-NW group showing a change of -2.64 cm [*P* = 0.014; Cohen’s *d* = -0.591; 95% CI: (-4.68, -0.61)] and the ST group demonstrating a change of **-**2.83 cm [*P* = 0.011; Cohen’s *d* = -0.601; 95% CI: (-4.86, -0.79)]. There was no significant difference in WC change between the HII-NW and ST groups (*P* = 0.952). Both groups exhibited significant increases in HDL-C levels after training, with the HII-NW group’s HDL-C increasing by 5.30 mg/dL [*P* = 0.007; Cohen’s *d* = 0.673; 95% CI:(1.72, 8.88)] and the ST group’s HDL-C showing an increase of 3.34 mg/dL [*P* = 0.016; Cohen’s *d* = 0.542; 95% CI:(0.68, 6.00)]. Moreover, significant decreases in FBG levels were observed in both groups after training, with the HII-NW group showing a reduction of 2.71 mg/dL [*P* = 0.019; Cohen’s *d* = -0.559; 95% CI:(-4.93, -0.50)] and the ST group showing a decrease of 2.22 mg/dL [*P* = 0.045; Cohen’s *d* = -0.444; 95% CI:(-4.38, -0.06)]. However, when compared to pre-training levels, a significant decrease of 13.95 mg/dL [*P* = 0.027; Cohen’s *d* = -0.551; 95% CI:(-25.49, -2.4)] in TG was only observed in the HII-NW group.

**Table 3:** Body measurement characteristics between groups pre and post 12 weeks of training.

Characteristics	Change within group (12 weeks- 0 weeks)			Comparison between 3 groups (12 weeks-0 weeks)
	HII-NW group (n=21)	ST group (n=23)	CG croup (n=21)	*P*
Primary outcome (change after 12-week intervention)				
WC, cm	-2.64 ±4.47 *	-2.83 ±4.70 *	0.00 ±1.70	*0.33*
SBP, mmHg	-2.22 ±13.38	-6.30 ±14.99	-4.56 ±10.66	*0.74*
DBP, mmHg	-1.19 ±8.95	-2.46 ±7.35	5.37 ±4.69	*0.16*
TG, mg/dl	-13.95 ±25.34 *	4.65 ±26.44	0.33 ±19.11	*0.35*
HDL-C, mg/dl	5.30 ±7.87 *	3.34 ±6.16 *	-0.13 ±3.26	*0.11*
FBG, mg/dl	-2.71 ±4.86 *	-2.22 ±5.00 *	5.52 ±9.38 *	*0.01†*
Secondary outcome (change after 12-week intervention)				
BMR, kcal/d	3.08 ±25.31	1.00 ±26.81	1.54 ±24.27	*0.77*
PBF, %	-0.76 ±1.66 *	-1.46 ±1.47*	0.03 ±0.74	*0.05†*
AOD	-0.03 ±0.02 **	-0.02 ±0.03 **	0.00 ±0.03	*0.24*
BMI, kg/m²	-0.19 ±0.45	-0.60 ±0.95 *	-0.07 ±0.60	*0.10*
ACT, min/w	20.68 ±22.83	12.64±2.06	-2.16 ±7.60	*0.63*
ANS, steps/day	466.48±174.54	193.98±196.79	-297.09 ±672.49	*0.60*
HR, bpm	-4.94 ±9.94 *	-2.84 ±10.12	-1.37 ±4.71	*0.28*
Non-HDL-C, mg/dl	-5.89 ±42.22	1.33 ±24.70	-7.79 ±34.64	*0.98*
LDL-C, mg/dl	-2.38 ±39.51	5.87 ±32.62	3.57 ±15.25	*0.86*

Data are expressed as mean (± SD); Pre, measurements at baseline; Post, measurements after 12 weeks of training; * significantly different from pre, P<0.05; ** significantly different from pre, P<0.01; † significantly different between groups, P<0.05.

HII-NW, High-Intensity Interval Nordic Walking; ST, Strength Training; CG, Control Group; BMR, Basal Metabolic Rate; PBF, Percent Body Fat; AOD, Abdominal Obesity Degree; ACT, activity time per week [min]; ANS, Average number of steps per day; BMI, Body mass index; WC, Waist circumference; SBP, Systolic blood pressure; DBP, Diastolic blood pressure; HR, Heart rate; TG, Triglycerides; HDL-C, High Density Lipoprotein Cholesterol; NO-HDL-C, non-High Density Lipoprotein Cholesterol; LDL-C, Low Density Lipoprotein Cholesterol; FBG, Fasting blood glucose.

**Figure 3:**
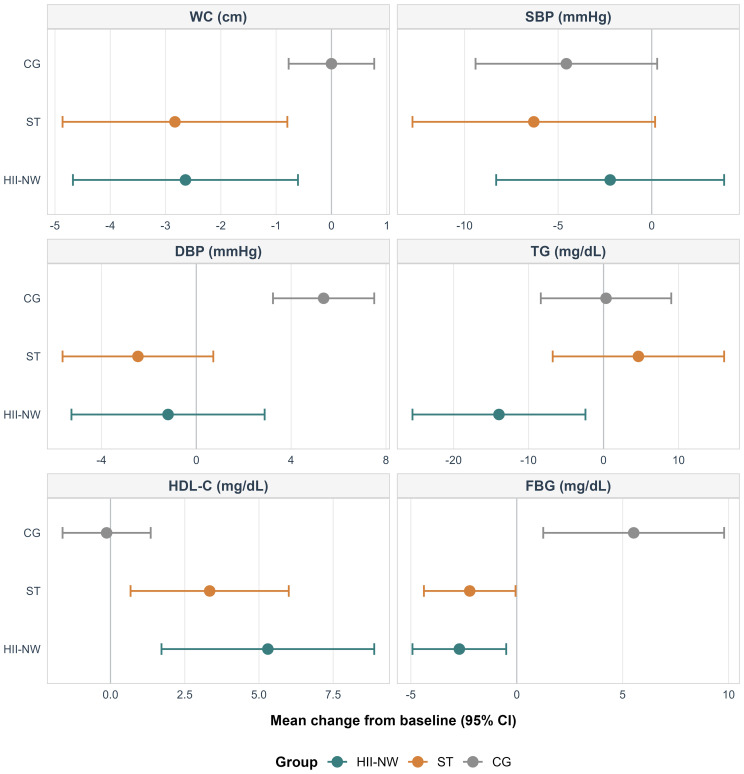
Comparison of primary outcomes measures between groups. HII-NW, High-Intensity Interval Nordic Walking; ST, Strength Training; CG, Control Group; WC, Waist circumference; TG, Triglycerides; HDL-C, High Density Lipoprotein Cholesterol; FBG, Fasting blood glucose.

A comparison of the changes in primary outcome indicators pre- and post-training is illustrated in **Figure 3**. After the training, the FBG levels in both the HII-NW group and the ST group were significantly lower than those in the control group, and there was no significant difference in FBG levels between the HII-NW group and the ST group (p = 0.431).

### Secondary outcome

**Figure 4:**
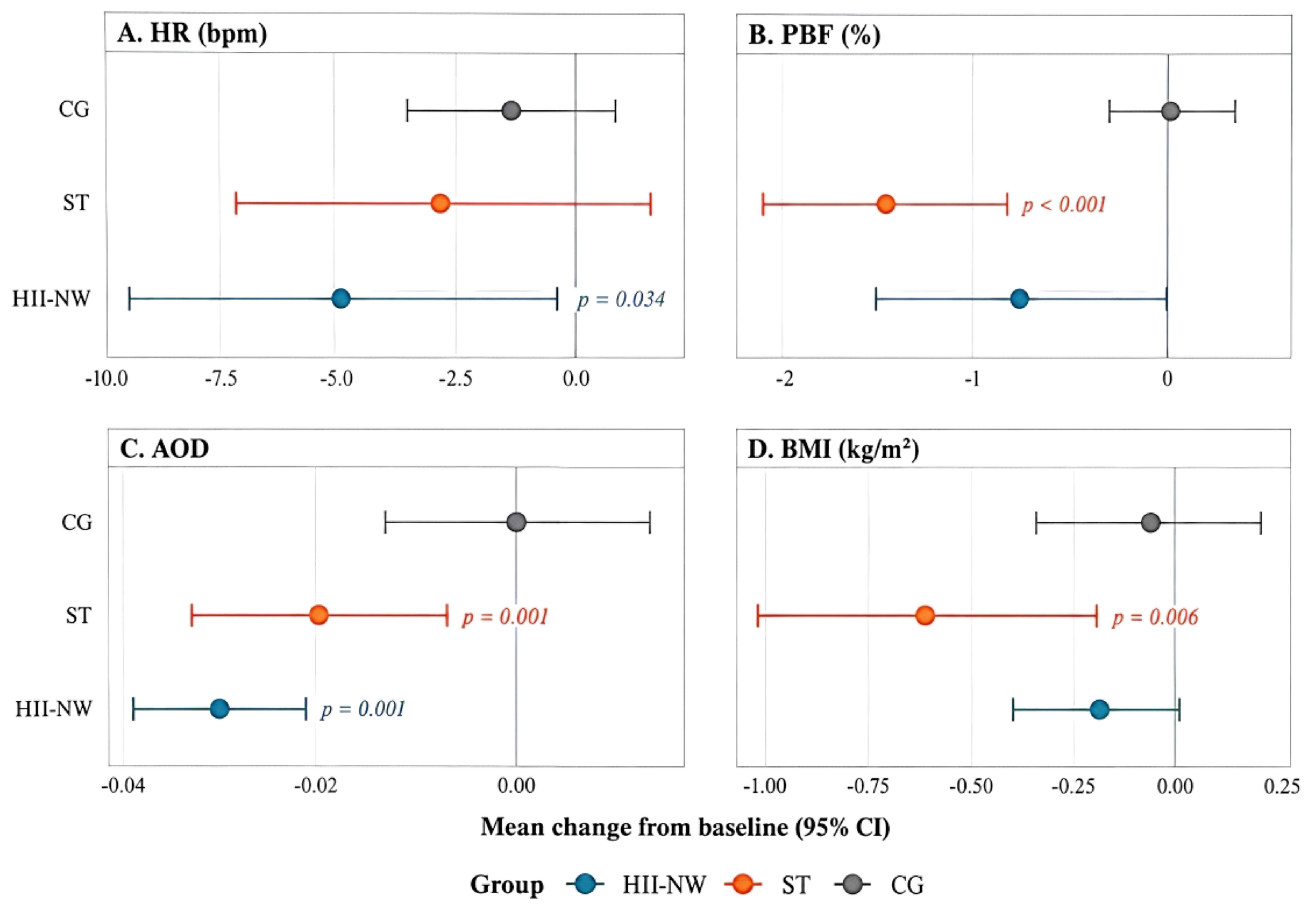
Comparison of secondary outcomes measures between groups. HII-NW, High-Intensity Interval Nordic Walking; ST, Strength Training; CG, Control Group; HR, Heart rate; PBF, Percent Body Fat; AOD, Abdominal Obesity Degree; BMI, Body mass index.

Following the 12-week exercise intervention, both HII-NW and ST training significantly reduced various obesity parameters among participants, including PBF, AOD and BMI. Specifically, HII-NW training resulted in a reduction of PBF by 0.76 percentage points [*P* = 0.05; Cohen’s *d* = -0.454; 95% CI:(-1.51, 0.00)], AOD by 0.03 percentage points [*P* = 0.001; Cohen’s *d* = -1.11; 95% CI:(-0.04, -0.02)]. In contrast, ST training led to a decrease in PBF of 1.46 percentage points [*P* = 0.000; Cohen’s *d* = -0.991; 95% CI:(-2.09, -0.82)], a reduction in AOD of 0.02 percentage points [*P* = 0.001; Cohen’s *d* = -0.779; 95% CI:(-0.04, -0.01)], and a decline in BMI of 0.60 [*P* = 0.006; Cohen’s *d* = -0.631; 95% CI:(-1.01, -0.19)]. Additionally, the HII-NW group exhibited a significant decrease in resting heart rate (HR) by 4.94 beats per minute [*P* = 0.034; Cohen’s *d* = -0.496; 95% CI:(-9.46, -0.41)]. No significant changes in basal metabolic rate (BMR) were observed across the groups pre and post training (p > 0.05). However, both the HII-NW and ST groups experienced significant increases in ACT and ANS when compared to pre-training levels (p < 0.05). The comparison of changes in secondary outcome indicators pre and post training for each group is illustrated in **Figure 4**. Inter group comparisons revealed significant differences in PBF among the three groups post-training, with paired comparisons indicating that the PBF of the ST group was significantly lower than that of the CG group (p < 0.05).

## Discussion

Exercise training plays a significant role in the prevention and management of various age-related diseases (Pedersen and Saltin, 2015), with particularly notable effects on MetS (Ingle et al., 2017). Previous research highlights inconsistent findings regarding how exercise modality and intensity influence MetS related biomarkers (Wewege et al., 2018). In this context, our 12-week quasi-experimental studies aimed to clarify the unique impacts of HII-NW and ST on obesity and MetS parameters in PW with abdominal obesity. Our core findings reveal that both interventions effectively reduced WC and improved lipid profiles (e.g., increased HDL-C, reduced FBG), with HII-NW demonstrating additional benefits in TG reduction and HR improvement. The core novelty of this study lies in that we directly compared HII-NW (a novel exercise modality optimized for PW) with ST, and clarified their differential effects on MetS biomarkers in abdominally obese PW. HII-NW overcomes the limitations of traditional aerobic exercise and standard HIIT, achieving a balance between low-impact safety and high metabolic stimulation, thereby providing a new option for precision exercise interventions in PW.

The present findings are largely consistent with our *a priori* hypotheses. Hypothesis 1 (H1) was confirmed: the HII−NW group showed significantly greater reductions in TG (−13.95 mg/dL, p = 0.027) and greater improvements in HR (−4.94 bpm, p = 0.034) compared with the ST group. These results support the metabolic benefits of combining Nordic walking with high−intensity interval training and align with our expectation that HII−NW would more effectively improve lipid profiles and cardiovascular−related indicators. Hypothesis 2 (H2) was confirmed: the ST group exhibited superior reductions in BMI (−0.60 kg/m², p = 0.006) and greater improvements in PBF (−1.46%, p < 0.001) compared with the HII−NW group, consistent with our expectation that ST improves metabolic health by increasing muscle mass. Abdominal obesity is a critical metabolic risk factor in PW, strongly linked to all-cause mortality and cardiovascular disease (Eguchi et al., 2012; Lera Orsatti et al., 2014). While global guidelines generally recommend endurance training for weight management (World Health Organization, 2020), the specific advantages of HIIT and resistance-based modalities like ST remain underexplored in this demographic. Unlike traditional aerobic exercise, HII-NW uniquely combines pole-supported walking with intensity intervals, which may enhance energy expenditure and recruit upper-body musculature, thereby amplifying fat oxidation (Mieszkowski et al., 2018; Micielska et al., 2021). This mechanism aligns with meta-analyses showing HIIT’s superiority in reducing visceral and abdominal fat compared to moderate-intensity continuous training (MICT), likely due to its ability to elicit excess post-exercise oxygen consumption and boost insulin sensitivity (Maillard et al., 2016; Wewege et al., 2017). For instance, Maillard et al. demonstrated that HIIT reduces abdominal fat mass in PW with type 2 diabetes, a benefit attributed to its time-efficient design (40% less training volume than MICT) (Maillard et al., 2016). ST, conversely, targets muscle mass preservation and metabolic rate elevation through progressive resistance. While ST alone may not induce significant weight loss, it promotes fat-free mass retention and enhances basal metabolic rate, as observed in our study’s BMI reductions (Ahtiainen et al., 2015; Kazemi et al., 2023). This aligns with research showing that 12-week resistance programs improve lean mass and reduce fat mass in older women, even without caloric restriction (Normandin et al., 2017; García-Pinillos et al., 2019). Notably, the ST group in our trial achieved comparable WC reductions to HII-NW, suggesting that muscle strengthening exercises can effectively target abdominal obesity through mechanisms distinct from aerobic energy expenditure, such as improved insulin mediated glucose uptake (Donnelly et al., 2009; Ahtiainen et al., 2015). By focusing on these modality-specific mechanisms, our study bridges a critical gap in understanding how HII-NW and ST uniquely address MetS in PW.

Consistent with Hypothesis 2 (H2), a 12 - week training program, in which participants in the HII - NW and ST groups engaged in training three times a week, could similarly reduce PBF, AOD, and BMI in PW with abdominal obesity. Notably, ST training induced a more pronounced reduction in BMI (-0.60 ± 0.95, p < 0.05) compared to HII-NW (-0.19 ± 0.45, p > 0.05), while both groups showed significant decreases in PBF (-1.46% ± 1.47 vs. -0.76% ± 1.66, respectively, p < 0.05 for both), This finding is associated with the mechanism by which ST promotes muscle fiber growth and elevates basal metabolism through progressive resistance training, further supporting our *a priori* hypothesis. The results of this study are in line with those of Maillard et al. (2016) and Mandrup et al. (2017). Their studies showed that HIIT could improve body composition in PW, such as reducing abdominal fat mass and improving body fat percentage. Steckling et al. (2019) reported that HIIT had no effect on overall fat in PW with MetS. However, it should be noted that this study did not assess dietary intake and/or physical activity levels during the intervention, which may have affected the results. Martin et al (Martins et al., 2018) also found that neither HIIT nor the combination of aerobic and resistance training had an impact on total body fat (%) and muscle mass index in PW after a 12 - week intervention. The differences in research results may be related to differences in study populations, intervention protocols, and control of confounding factors. Davidson et al. (2009) found that in elderly obese adults, a 6 – month MICT combined with RT program led to greater overall, abdominal, and visceral fat loss compared to MICT or RT alone. This suggests that combined training may have a more significant effect on reducing body fat, which is consistent with the trend that both HII - NW and ST showed good effects on reducing obesity indicators in this study. In terms of ST, the results of this study are consistent with previous evidence on ST interventions in older women. A 12 - week resistance training program can increase lean mass and muscle quality while reducing weight, fat mass, and BMI (García-Pinillos et al., 2019). Moreover, additional evidence emphasizes that both the volume of resistance training (Morton et al., 2019) and HIIT are crucial factors influencing muscle quality response and leading to reduced BMI.

WC, an indirect indicator of abdominal fat, serves as a key criterion for diagnosing MetS and is closely linked to the health of older women. Consistent with Hypothesis 1 (H1), our 12-week study revealed significant WC reductions in both the HII-NW and ST groups. These reductions were associated with improved metabolic parameters, including increased HDL-C and reduced FBG in both training groups. The present study found that HII-NW significantly reduced TG levels (−13.95 mg/dL, p = 0.027), whereas no significant change was observed in the ST group. This result suggests that HII-NW may activate the PPAR-α signaling pathway (Normandin et al., 2017; Campbell et al., 2019) through the combined effects of pole-assisted Nordic walking and high-intensity intervals, thereby promoting hepatic TG catabolism and TG uptake in peripheral tissues. This mechanism differs from standard HIIT, which regulates lipid metabolism primarily through the AMPK pathway, and represents a novel finding of this study. Based on our results, we propose that HII-NW may achieve more precise regulation of lipid metabolism via the coordinated actions of PPAR-α and AMPK pathways, providing a new direction for future mechanistic studies of HII-NW. HIIT promotes visceral fat loss and adipocyte mitochondrial biogenesis through enhanced lipolysis (Boutcher, 2011; Mandrup et al., 2017), which is consistent with the reductions in WC and PBF observed in the present study. Intermittent high-intensity stimulation improves endothelial function and vagal tone (Greeley et al., 2013; Reljic et al., 2022), explaining the beneficial changes in HR and TG in the HII-NW group. Inconsistent findings across HIIT studies may stem from methodological limitations (e.g., no dietary control (Steckling et al., 2019)) or population differences (e.g., severe comorbidities (Martins et al., 2018)). The intensity of 75–85% HRmax used in our protocol optimizes fat oxidation (Boutcher, 2011; Wewege et al., 2017), while the pole-supported design of HII-NW enhances upper-body muscle engagement and reduces joint loading. These features may explain the superior effects of HII-NW on TG and HR compared with ST.

In our study, ST training significantly improved WC, HDL-C, and FBG levels in PW。Meanwhile, the ST group exhibited a significantly greater reduction in BMI relative to the HII-NW group. This finding is consistent with the well-documented mechanism whereby ST increases muscle mass and elevates basal metabolic rate. Notably, a significant correlation was observed between BMI reduction and decreased PBF in the ST group. This suggests that ST may lower BMI by augmenting muscle mass to enhance basal metabolism, which in turn facilitates lipolysis and fat breakdown. This correlative relationship has not been clearly reported in previous studies focusing on PW. Accordingly, the weight-regulating effect of ST in abdominally obese PW appears to be achieved through targeted optimization of body composition. However, ST did not significantly impact BP, HR, TG, or LDL-C. These results align with Normandin et al.’s findings (Reljic et al., 2022), which suggest that resistance training primarily influences weight-related metrics rather than comprehensively modulating all MetS biomarkers. Notably, HII-NW training demonstrated broader efficacy in improving MetS biomarkers. This may be attributed to its unique integration of HIIT and Nordic walking elements: The intermittent high-intensity stimulus (75–85% HRmax) enhances catecholamine-driven lipolysis and mitochondrial biogenesis in adipose tissue, leading to more pronounced reductions in TG and abdominal fat (WC) (Greeley et al., 2013; Mandrup et al., 2017). Additionally, HIIT improves endothelial function and vagal tone, explaining the significant HR and BP improvements in the HII-NW group (Wewege et al., 2017,58). NW-specific biomechanics, the use of poles during NW engages upper-body muscles and promotes whole-body movement, increasing energy expenditure during exercise. This dual focus on aerobic capacity and full-body coordination may amplify metabolic stress and lipid metabolism, as reflected by the HII-NW group’s greater TG reduction (Mieszkowski et al., 2018; Micielska et al., 2021). While both interventions improved key MetS markers, HII-NW’s superiority in TG, HR, and BP regulation highlights the synergistic advantages of combining interval intensity with weight-bearing, bilateral limb movement. Under the conditions of this study, HII-NW showed potential advantages in improving multiple MetS parameters, suggesting it may be a promising comprehensive strategy for this population, but further validation is needed. There was one unanticipated finding in the present study: no significant between-group differences were observed in improvements in SBP and DBP between the HII-NW and ST groups (p > 0.05). This outcome partially diverged from our initial expectation that HII-NW would yield greater improvements in cardiovascular indicators. The lack of significant differences may be attributed to the relatively small sample size, the 12-week limited intervention duration, and uncontrolled confounding factors such as dietary habits. Further verification with more rigorous design is warranted in future investigations.

### Strengths and limitations

This study has several notable strengths. First, the research design is scientifically rigorous and highly targeted. A quasi-randomized controlled trial design was employed, along with internationally recognized tools such as the InBody 720 body composition analyzer and Polar V-800 heart rate monitor, and standardized procedures. Over 10 core indicators of MetS, including WC, blood lipids, FBG, and BP, were comprehensively monitored in PW with abdominal obesity who met ≥2 MetS criteria. This high-risk population has been underrepresented in previous research, and this study fills an existing gap in the field. Second, the study innovatively compared the intervention effects of two exercise modalities. As the first study to directly compare the impacts of HII-NW and ST on MetS in PW, it revealed that HII-NW was more effective in reducing TG, lowering heart rate, while ST had a more significant effect on reducing BMI. These findings provide direct evidence for personalized exercise intervention selection, breaking through the traditional paradigm dominated by aerobic exercise. Third, the clinical application value is evident. The study confirms that both training modalities can effectively improve core MetS biomarkers, such as WC, HDL-C, and FBG. Clinicians can formulate exercise prescriptions based on these results, which helps in reducing the risk of cardiovascular diseases in this population.

Despite its rigorous design, the study has several limitations. First, inadequate control of confounding factors: Baseline variables such as age, BMI, and the use of antihypertensive and lipid-lowering medications were not included as covariates in the model. Although analysis of covariance was used to control for baseline effects, the lack of clear reporting on whether these variables were included may have led to residual confounding. Future research should use multivariate regression for systematic control. Second, limited sample size and intervention duration: Only 21–23 participants completed the 12-week intervention in each group. Although the sample size was pre-calculated, small samples may fail to detect minor but clinically meaningful differences, and the short intervention period may not fully capture long-term metabolic changes. Third, potential bias in randomization: Quasi-randomization based on geographical location, while controlling for environmental variables, may have introduced bias related to socioeconomic status. The lack of stratified analysis of relevant indicators affects the sample representativeness. Fourth, the study did not utilize a standardized questionnaire to monitor adverse events, which may limit the comprehensive assessment of intervention safety. Nevertheless, participants were regularly asked about their health status by the supervising trainers, both during sessions and in cases of absence, constituting a basic form of safety monitoring. Future research could increase the sample size to ≥50 participants per group, extend the intervention period beyond 6 months, adopt stratified randomization combined with dynamic monitoring of confounding factors, and incorporate nutritional interventions to evaluate synergistic effects, formal and validated tools for adverse event assessment should be considered for implementation to ensure greater reliability of the collected data. This study adopted a quasi-experimental design and did not strictly control potential confounding factors such as diet and daily physical activity, which may lead to biases in the interpretation of some results. Therefore, the “advantages” mentioned in the conclusions of this study only represent inter-group associations rather than definite causal relationships, which need to be further verified in subsequent studies through strict control of confounding factors.

## Conclusion

Within the limitations of this 12-week quasi-experimental study, HII-NW was associated with significant reductions in TG levels (p<0.05), PBF, and resting heart rate, while ST was associated with a significant reduction in BMI; these differences suggest potential advantages of each intervention, but cannot be interpreted as definitive causal effects due to uncontrolled confounding factors (e.g., diet, daily physical activity. Future studies with larger samples, longer interventions, and comprehensive confounding controls (e.g., dietary intervention) are needed to validate these results, confirm potential causal relationships, and explore long-term effects.

## Data Availability

The datasets presented in this study can be found in online repositories. The names of the repository/repositories and accession number(s) can be found in the article/**Supplementary Material**.
