# Supplementary material for: Effect of high intensity interval Nordic walking and strength training on selected biomarkers of metabolic syndrome in postmenopausal women with abdominal obesity: a quasi-experimental studies
Source: Front Physiol. 2026 Jun 19;17:1841217. doi: 10.3389/fphys.2026.1841217 (PMC13327994; doi:10.3389/fphys.2026.1841217)
Supplement: Supplementary file 1 [file Table1.docx]

**Table.S1** Number of people meeting indicators of MetS in each group– IDF criteria

|  | HIIT NW（n=21） | ST（n=23） | CG（n=21） | Total |
| --- | --- | --- | --- | --- |
| WC | 21 | 23 | 21 | 65 (100%) |
| BP | 14 | 18 | 15 | 47 (72.3%) |
| FBG | 9 | 14 | 12 | 35 (53.9%) |
| TG | 7 | 7 | 7 | 21 (32.3%) |
| HDL-C | 9 | 7 | 9 | 25 (38.5%) |

Abbreviations

WC: Waist circumference; BP: Diastolic blood pressure; FBG: Fasting blood glucose; TG: Triglycerides; HDL: High Density Lipoprotein Cholesterol;
